# Complete Genome Sequence of *Polynucleobacter* sp. Strain TUM22923, Isolated from Antarctic Lake Sediment

**DOI:** 10.1128/mra.00146-23

**Published:** 2023-06-21

**Authors:** Sho Shimada, Ryosuke Nakai, Kotaro Aoki, Shota Miura, Kohji Komori, Sakae Kudoh, Satoshi Imura, Yoshikazu Ishii, Kazuhiro Tateda

**Affiliations:** a Department of Microbiology and Infectious Diseases, Toho University School of Medicine, Tokyo, Japan; b Department of Respiratory Medicine, Tokyo Medical and Dental University (TMDU), Tokyo, Japan; c Bioproduction Research Institute, National Institute of Advanced Industrial Science and Technology (AIST), Sapporo, Japan; d National Institute of Polar Research, Research Organization of Information and Systems, Tokyo, Japan; e Department of Polar Science, The Graduate University for Advanced Studies, SOKENDAI, Tokyo, Japan; University of Delaware College of Engineering

## Abstract

Here, we report the complete genome sequence of *Polynucleobacter* sp. strain TUM22923, isolated from Antarctic lake sediment. This strain has a genome of 1,860,127 bp, comprising 1,848 protein-coding sequences. These sequence data could contribute to the elucidation of genome streamlining and low-temperature adaptation in members of *Polynucleobacter*, a cosmopolitan group of ultramicrobacteria.

## ANNOUNCEMENT

Members affiliated with subcluster PnecC of the genus *Polynucleobacter* have a cosmopolitan distribution in freshwater systems (1, 2). They are characterized by ultrasmall cells and are accordingly defined as ultramicrobacteria (cell volume, <0.1 μm^3^ [3]). Here, we report the complete genome sequence of a *Polynucleobacter* bacterium, TUM22923, isolated from Antarctica. Strain TUM22923 was isolated from a freshwater lake, tentatively named Lake Nishi-yukidori Ike (formerly known as Lake Kitanotani Ike; 69.2369 S, 39.7381 E [4]), in the Langhovde ice-free area, East Antarctica. The lake water temperature at the time of sample collection (December 2018) was 2.6°C, pH was 8.69, and conductivity was 103.5 μS/cm (4). Fifty grams of lake sediment containing lake water was added to 100 mL of sterile distilled water and incubated at 4°C for 4 months. The suspension supernatant was spread onto R2A agar medium (Becton, Dickinson and Company, Franklin Lakes, NJ) and incubated at 15°C for several weeks. The resulting strain, designated TUM22923, was purified by single-colony isolation.

For genome sequencing, strain TUM22923 was cultured for 4 weeks on R2A agar medium at 15°C. Genomic DNA was extracted using magLEAD 6gC combined with magDEA Dx SV (Precision System Science, Chiba, Japan). Whole-genome sequencing was performed using MiSeq (Illumina, San Diego, CA, USA) and MinION (Oxford Nanopore Technologies, Oxford, UK) instruments. DNA for MiSeq sequencing was prepared using Illumina DNA Prep (M) Tagmentation, IDT, for Illumina DNA/RNA Unique Dual indexes, and MiSeq reagent kit v3. The MiSeq reads were paired-end reads of 350 bp (read 1) and 250 bp (read 2). MinION sequencing was performed using a rapid barcoding kit (SQK-RBK004) and R9.4 flow cell (Oxford Nanopore Technologies). Base calling was performed using Guppy version 6.3.8. The number of raw reads by MiSeq and Nanopore sequencing were 875,875 and 10,062, respectively. The *N*_50_ length of Nanopore reads was 16,675 bp. MiSeq and MinION reads were quality filtered and adapter trimmed using Trimmomatic tool version 0.39 (quality score cutoff of 20) (5) and NanoFilt version 2.8.0 (quality score cutoff of 6 and minimum length 5,000 bp) (6), respectively. Qualified reads were subsequently used for hybrid assembly using Unicycler version 0.5.0 (7), and the contigs obtained were polished three times using Pilon version 1.24 (8). Unless otherwise stated, default parameters were used for all software. The assembly data were processed using the DFAST annotation pipeline (version 1.2.18) incorporating quality assessment using average nucleotide identity (ANI) (9). The full-length 16S rRNA gene sequence obtained was searched with nucleotide BLAST (10) against the NCBI nucleotide/nonredundant database (accessed January 2023).

The complete genome sequence of strain TUM22923 was obtained by hybrid *de novo* assembly. The genome comprises 1,860,127 bp in total, a G+C content of 46.1%, 1,848 protein-coding sequences, 3 rRNA genes, and 40 tRNA genes. The average sequence depth was 74.8×. The 16S rRNA gene sequence of strain TUM22923 was completely identical to that of Polynucleobacter brandtiae strain UB-Domo-W1^T^ belonging to the PnecC lineage (DDBJ/ENA/GenBank accession no. MK926443.1). Strain UB-Domo-W1^T^ was isolated from a polar region, specifically Lake Domo, Byers Peninsula, Maritime Antarctica (2). However, their ANI value was low at 91.3%. This discrepancy may reflect cryptic diversity that cannot be resolved based on 16S rRNA gene sequence analysis, as shown previously (11). The genomic data will be essential for elucidation of genome streamlining associated with small genome size, as well as low-temperature adaptation among the widely distributed PnecC bacteria.

### Data availability.

The complete genome sequence of strain TUM22923 has been deposited in the DDBJ/ENA/GenBank database under accession no. AP027274.1 (BioProject/BioSample no. PRJDB14135/SAMD00575312) and DDBJ Sequence Read Archive no. DRR439343 and DRR439344.
